# Awareness of Patient Physical Handling Issues Associated with Routine Nursing Care

**DOI:** 10.3390/nursrep10020009

**Published:** 2020-10-16

**Authors:** Marie Trešlová, Drahomíra Filausová, Lenka Šedová, Hana Hajduchová

**Affiliations:** Faculty of Health and Social Science, University of South Bohemia in České Budějovice, 37005 České Budějovice, Czech Republic; filausov@zsf.jcu.cz (D.F.); lsedova@zsf.jcu.cz (L.Š.); hajducho@zsf.jcu.cz (H.H.)

**Keywords:** physical handling, quality, safety, patient, nursing staff, management

## Abstract

Introduction: Physical handling (PH) of patients is an essential component of nursing care. It is an intervention that is troublesome for patients and strenuous for general nurses and other nursing staff. Handling techniques and mechanical aids for PH have improved through the years; however, they are not routinely used in nursing practice. Aim: The aim of this research was to determine (1) the level of awareness of PH issues within the management of South Bohemian hospitals, (2) how management perceives, organizes and implements PH protocols, and (3) how PH protocols are applied in everyday nursing practice. Method and Research Sample: Two qualitative methods were used: a semi-structured interview and observation. The participants were management representatives of South Bohemian hospitals in Czech Republic. Fifty nurses were observed during PH at the same hospitals. Results: The study found that PH was not monitored, nor was it provided systematically. In spite of this, no serious drawbacks were found; nonetheless, many areas were identified where the quality of PH could be improved. For example, awareness of PH issues by management, educational programs teaching new handling techniques, and provision of state-of-the-art PH aids. Several issues related to PH were found to be related to staff shortages, which remains a major problem. Conclusion: This issue needs more attention; it should be addressed by both those doing nursing research as well as those engaged in everyday nursing practice. Both will require the assistance of hospital staff and management.

## 1. Introduction

Physical handling (PH) of patients during nursing care is a basic skill required of all nursing staff and has developed through the centuries. Many present-day research studies have started looking at this topic, e.g., Lee et al. [1]. These articles show that the issue of physical handling is a neglected research topic in Czech Republic. In contrast, foreign authors have investigated PH risk prevention from various aspects such as staff numbers, quality of care, various kinds of aids, patient condition, types of hospital departments, education, staff injuries, role of management, etc. [2,3,4]. One of the characteristics of high-quality care is patient satisfaction and nurse satisfaction, which is influenced by working conditions [5,6]. High-quality and professional nursing care is based on evidence provided by research (EBN) [7]. The essential question for EBN is: “Is the work we are doing the most suitable for the health and wellbeing of the patient?” Within the hospital organization, quality of care is controlled by hospital management. The WHO [8] defines high-quality care as the sum of the results achieved in prevention, diagnosis, and therapy determined by the needs of the population based on medical sciences and practice. As early as 1986, Donabedian [9] described quality care as care that makes the greatest contribution to the patient´s health and the achieved benefit, in comparison with the costs, is higher than in all phases of the treatment process. It takes into account not only the outcome, accessibility, equity but also the safety of care. Qvretveit in McMillan et al. [10] defines it as the ability to meet the needs of those who are dependent on the care. It is the ability to restore function, eliminate pain, prolong a productive or meaningful life, answer questions, and respect human dignity. The WHO [11] renews the original definition and emphasizes the relationship between quality care and contemporary knowledge and technical development. It also describes barriers to the provision of high-quality care that include: inappropriate training, failure to meet quality standards or the absence of such standards, inappropriate equipment, time stress, insufficient staff, fatigue, and incurred costs. According to the above-mentioned definitions, the issue of physical handling is undoubtedly a potential risk that needs to be eliminated in order to ensure high-quality care. However, it is related to both the price and accessibility [9]. 

To ensure greater patient comfort and to reduce the physical demand on nursing staff, a number of modern PH aids and devices have been developed [12]. In order to use these aids and devices effectively, staff needs to undergo training (both in nursing school and as continuing professional education) on how to use new handling techniques [13]. The management of health care facilities must be willing to invest in the purchase of new equipment and associated training. The motivation for such investments can be seen by monitoring not only the negative effects of inappropriate physical handling (patient injuries, staff injuries) [14] but also the positive benefits that come from preventing injuries [15,16]. The cost of lost staffing due to on-the-job injuries [17] can easily exceed the cost of new equipment [9]. Additionally, patient injuries caused by a lack of equipment or staff training can lead to legal liability, the cost of which can be considerable [18]. Currently, the methodology of adverse events monitoring [19] does not consider musculoskeletal injuries incurred during normal nursing duties to be an adverse event. We see the differences between international and Czech nursing education and practice. Nursing in the Czech Republic still strives to achieve the credit of a well-developed and EBP discipline. The purpose of the study was to determine the status and the situation of PH in a monitored hospital to be able to propose and organize courses to improve PH in practice. Therefore, our research question was: What awareness of patient physical handling issues does the hospital management have and how does it assures the quality and safety of patients and nursing staff connected with physical handling?

## 2. Methods

The top management of five hospitals in the Czech Republic’s South Bohemia region participated in a qualitative research investigation implemented using semi-structured interviews. These hospitals are our faculty’s active collaborators in several researches. There is an effort to raise the quality of care on the faculty as on the hospital’s side. These hospitals are also our clinical placements for students’ clinical practice. Answers were recorded in prepared record sheets, and then analyzed using the paper and pencil method, to code and categorize transcribed text. During preparation of the research drafts, we created five categories for investigation on the bases of literature study: (1) documents; (2) form and system of quality and safety; (3) dealing with complaints and management; (4) perception and organization of physical handling by the management and education; (5) safety, risk and management. We also used hidden observers in the above-mentioned hospitals to confirm results. In the pilot phase, 5 observations from each department were required from each hospital. The following departments were observed: the internal medicine department, the surgical department, the anesthesiology-intensive care department, and the subsequent care department. We received 50 observation sheets back to analyze. Only physical handling interventions (PHI) performed by nurses were used as a selection criterion. The following PHI were monitored: (1) sitting up in bed, (2) sitting on the side of bed with legs dangling, (3) repositioning in bed, (4) changing bed linen, (5) hygiene done in the bed, (6) lying down from a sitting position, (7) transfer from bed to chair/wheelchair and back, (8) hygiene from bed to bath, (9) standing up from a wheelchair, (10) standing up from the bed. During observations, observers specified the number of PH interventions, which could consist of multiple interventions, e.g., hygiene in the bed (5 times) and changing bed linen (4 times). The observers were unit nurses addressed by head nurses out of each individual hospital. They had been instructed about the observation sheet and how to use the observation scale. Observers worked together with shift nurses and monitored one nurse during provision of one FM. The observation sheet used to record PH interventions was developed based on theoretical knowledge and the study of literature resources. It included the most common nursing interventions associated with PH, the level of patient self-sufficiency, risk of patient injury, use of aids, comfort, and the degree of communication and cooperation with the patient. There was patient expression of possible pain, discomfort and fear observed and recorded. Nurses were also monitored during PH interventions, i.e., preparation of the environment and the patient for physical handling, use of good body mechanics by the nurse, utilization of efficient physical handling techniques to prevent musculoskeletal injuries to the nurses, and whether the nurse used effective communication to ensure the security and safety of patients during the PH intervention. For each item of observation, the two evaluation systems were used—either pass-fail or a three-stage score (positive, partial, negative). Observations were done by nurses working in the specific departments, which included both staff nurses, charge nurses, and/or nurse managers. Due to the current shortage of nurses, many of the nurses in the above-mentioned positions also regularly participated in nursing care. To respect the anonymity of individual hospitals, we do not specify the results in relation to particular hospitals.

All subjects gave their informed consent for inclusion before they participated in the study. The study was conducted in accordance with the Declaration of Helsinki, and the protocol was approved by the Ethics Committee of University of South Bohemia.

## 3. Results and Discussion

Table 1 shows the most worthwhile mentioned answers out of the five categories.

### 3.1. Category 1: Documents

Ensuring care is defined in [16,20,21,22]. Therefore, we tried to determine which of the documents that provide foundational information on the physical handling of patients in nursing care were used by the hospitals involved in our research. Except for one hospital that stated that it “does not have anything like that,” the other hospitals unanimously cited: “Standards, Standard Procedures of Nursing Care or Guidelines.” Although it was not specified in detail how physical handling was addressed by these documents. However, according to the statements, these documents are related to “rehabilitation nursing, manual handling of loads, risks assessment with the focus on falls, safety and health protection at work, keeping health and nursing documentation, evaluation of patient´s self-sufficiency, records of patient positioning, and use of restraining devices.” They also cited concrete laws and regulations: “e.g., Regulation of the Government determining conditions of occupational health protection, Law on provision of other conditions of safety and health protection at work, Decree on prohibited work and workplaces (pregnant women, juvenile workers.” One hospital even cited the European Occupational Safety and Health Administration—EU-OSHA. One hospital gave a simple answer, that: “We do not have a standard course of action; we proceed according to the actual situation. We do not act based on foreign documents.” On the basis of the above-mentioned responses, we assumed that attitudes toward physical handling are not the same in all the hospitals and that the issue is not approached systemically. The research studies elaborated by Mayeda-Letourneau [23] and also the educational documents provided by Krill et al. [24] and Fitzpatrick [25] testify, that in other countries the issue of PH is a very important topic that is supported by multiple lines of evidence [26,27,28], and which is addressed on the grounds of financial expenses, quality of care and satisfaction of the employees, as well as staff turnover due to physical load.

### 3.2. Category 2: Form and System of Quality and Safety

If we wanted to monitor the quality and safety of care, it was necessary to define it and to develop a standard by which staff would be guided and motivated by management to comply8]. Compliance with the above-mentioned rules is monitored by the management, both visually and through documentation, using checks and audits [29]. Fitzpatrick [25] offers an overview of procedures—specification of the number of persons, equipment, and space requirements—which should be used by the staff to perform physical handling with regard to the physical and psychological state of the patient, his/her body weight and the potential for cooperation. This information from the assessment scales is part of the patient´s documentation, which ensures continuity of care with nurses on the next shift, thus preventing harm to the patient and staff in subsequent PH interventions. Therefore, we asked the question: “In what form or in what way is the information about patient PH in nursing care recorded?” One answer was that “it was not included” in the documentation. The other hospitals describe the form and the style of information about physical handling in various ways: “The record in nursing documentation with the nurse´s or physiotherapist´s signature; keeping of a positioning record; assessment of self-sufficiency of the patient at admission and any change of the patient´s condition during the anamnestic phase of the nursing process.” The positioning record is an indispensable part of nursing documentation [30,31], which specifies time limits for changing a patient´s position to prevent bedsores and thus, prevention of adverse events. Most often, this means only changing the lying position in bed, not changes from the lying to sitting position or transferring from the bed to another place. As such, the positioning record does not include information on the number of persons and aids needed for PH as described, for example, by Fitzpatrick [25,32]. High-quality and safe care, including PH, can only be provided by competent health care staff [24]. This was confirmed by statements from the participating hospitals claiming that they provide information to the staff in the form of “printed documents with pictures, organize working days including instructional videos concerning PH; and within regular training courses on safety and protection of health at work provide oral information dealing with the handling of loads.” The facilities participating in the research monitor the state, effects, and consequences of physical handling using: “reporting and evaluation of adverse events (falls, pressure ulcers); internal questionnaires investigating patient satisfaction; monitoring of compliance with the standards; evaluation of the quality indicators; anticipating hazards; evaluation of established preventive measures; introduction of corrective measures.” From the point of view of the employee, physical handling is included in reporting an employee´s injury connected with patient handling. One of the hospitals perceived PH as part of quality care and expressed its concrete position: “Monitoring of quality and safety concerns patient safety during handling and nursing care. One facility responded that PH “is not included” in their system of quality and safety. However, of the hospitals addressed, no one explicitly mentioned any document on PH. Based on the above-mentioned, we can say that PH is an intensively monitored issue, especially abroad. Very efficient methods for monitoring the quality and satisfaction involves feedback, which can be obtained from patients using forms/questionnaires aimed at evaluation of patient satisfaction and during supervision or reflective practice [33] with employees [23,34]. These statements, experiences and views can lead to changes—to improved quality of care and better work conditions. It can also lead to a reduction in employee turnover caused by dissatisfaction due to overload or work-related health issues [35].

### 3.3. Category 3: Dealing with Complaints and Management

The hospitals addressed did not have to deal with patient complaints about physical handling very often: “Complaints are rare and usually unjustified. It concerns especially excessively obese patients when there are problems with handling, adjustment, position changes and transfer where it is necessary to exert greater force. The situation is even worse when the patient does not cooperate or refuses to change position.” Some hospitals reported no problems with complaints: “We have not had such experiences. We did not register any complaints like that”. The complaints and concerns from staff are also infrequent. “We have not solved any official complaint of a staff member about the difficult physical handling of a patient so far.” However, if there were complaints, then “the complaints of the staff about difficulties and overloading during the physical handling of patients are often resolved by the department of subsequent care or by the internal, surgical, and neurological departments”. The statement that “a number of staff members who worked, for example, in a retirement home before their employment here in our health care facility regard the physical load during patient handling to be more easily managed” was found to be noteworthy. Based on the information from the hospitals involved, the reason for complaints is the “lack of auxiliary staff (health care assistants) and lack of lifting equipment and aids”. The management solves potential objections “verbally and by reporting them to the personnel department, objections are dealt with by middle management and handed over to hospital management”. Similar obstacles were reported by Constantin et al. [36]. The number of complaints about the physical handling of excessively obese patients is growing, and “because the hospital is not yet fully equipped with aids for this category of patients, we have only purchased a wheelchair for overweight patients. This is also a problem with regard to operating tables and tables for various examinations (CT, X-ray), etc. We do not have a systematic solution yet”. The issue of excessively obese patients is a global problem and is dealt with in many research studies [37,38].

### 3.4. Category 4: Perception and Organization of Physical Handling by Management and Education

Working conditions for the implementation of PH are characterized by education, suitable space, specific aids, and equipment [17,39,40]. We studied how management perceives and organizes physical handling. Management realizes that during PH, the musculoskeletal system of the staff is strained, which can lead to musculoskeletal injuries, and disorders; such injuries and disorders can lead to work absences. Inappropriate handling also increases the risk of harming the patient [6,38,41]. The hospitals involved in our research listed several aids and pieces of equipment that are routinely available or can be ordered according to the requirements of individual departments. We found that some hospitals have a wide range of educational events, sometimes even periodically repeated and dealing with topics going beyond physical handling itself. They seek to influence physical handling and quality of care and thus, can contribute to the implementation of professional nursing. Hospital managers ensure high-quality PH by providing aids and equipment: “They completely equip all departments with adjustable electrically-controlled beds, arrange for adaptation of rooms, bathrooms and toilets, equip the departments with mobile bathtubs, hoists, sliding sheets (in sufficient numbers) and mechanical patient lifts, ensure sufficient numbers of staff (including auxiliary staff), and few arrange installation of the Erilens ceiling lift systems”. Another answered that the provision of physical handling of patients in nursing care is only “restricted by the purchase of aids for lifting in selected departments”. Education of the staff is ensured by the management through “familiarizing employees with the standards and regulations; periodic training on safety and protection of health at work—using safe footwear familiarization with operating instructions for individual aids”. Notable was the information about the introduction of training courses focused on communication connected with PH: “specific training courses—communication skills”. Efficient communication, in addition to equipment, education, and functioning management, is an indispensable skill that needs to be used by nurses during physical handling. It is intended to calm patients and to improve cooperation. Patients should be given clear information before beginning a PHI, i.e., the nurse tells the patient what will be done, when, and how it will be done and who is going to do it, including asking the patient for cooperation when it is possible [38,42]. Other information was offered as “lectures—Back pain, one of the most frequent causes of morbidity, Save your back” and practical training led by the rehabilitation department. Not all employees take part in the regular, once a year, training—in contrast, for example, to periodic training in CPR. According to the HM statements, not all employees are interested in training and lectures on PH. They noted that “only employees who are interested in these lectures, and want to listen to them, apply and subsequently attend them”. Research studies, for example, Olkowski, Stolfi [43], Pirschel [44], and Theis and Finkelstein [3], provide evidence regarding the efficiency of staff training.

### 3.5. Category 5: Safety, Risk, and Management

To maintain or improve quality and safety, it is necessary to regularly monitor these parameters. This is done by using audits with subsequent remedial activities—education [45], the innovation of aids and equipment for physical handling, and assessments. Undertaking prevention and increasing risk awareness is more effective than dealing with the consequences [40]. The hospitals included in our research are aware of the risks and had implemented activities aimed at decreasing or eliminating risks: “Incorrect handling also increases the risk of harming a patient”. Safe care is also monitored “within the framework of the prevention of occupational diseases and absence due to sickness. “Staff safety during PH of patients in nursing care is ensured by HM: “audits of the documentation aimed at categorization of patients and the level of their self-sufficiency; plans of care and patient positioning records; purchase of technical lifts, which were not appraised positively by the staff because they are time-consuming. The functionality of the aids is regularly checked and maintained. Charge nurses and nurse managers of the individual departments address hospital management with requests for the purchase of lifting equipment. Based on the answers, we can ask whether all HM participants defined PHI in the same way. Some respondents focused only on positioning in the bed aimed at preventing pressure sores; others understood the term to apply to a whole spectrum of activities. It was apparent that PH is not included in systematic monitoring in all participating hospitals. This was confirmed by one of the answers: “This issue is not systematically monitored, mapped, or controlled”. 

A comparison of results from the two research techniques used in the investigation revealed that the attitudes of both the nurses and HM, relative to the performance of PHI, are identical. In our investigation, we tried to determine which nursing interventions require PH of patients. Out of nine suggested nursing interventions, PH was most often observed during the making of beds and changing bed linen, and during hygiene in bed. It was interesting that the observers did not record PH during patient positioning, in spite of the fact that HM repeatedly mentioned positioning records relative to documentation. We noted that many patients had restricted independence and therefore required in-bed hygiene care. We assume that as part of bed care for patients—lying patients—it is necessary to include these patients in the positioning schedule. Of 50 monitored patients, 25 were dependent on the nurse and 12 patients were unable to participate in the PHI at all; this included 8 patients who were unconscious. The observers evaluated the care provided by nurses as comfortable; PH caused pain in 15 out of 50 cases. Is it possible to consider such care as careful, even if it was believed by the observers to be careful on the basis of the observed elements? On the basis of the observation of nurses during the PH of patients, we can conclude that the nurses followed the basic rules of PH necessary for the safety of the patient and their own safety. These rules can be found, for example, in the documents “Moving and Handling” by Royal College of Nursing [46] as well as Fitzpatrick [25] and MacGregor [47] During the observations, we noticed that nurses adjusted the height of the bed, prepared patients for physical handling by providing information and describing what was to be done, prepared the patient´s body for subsequent easier handling, and prepared the environment to ensure safe handling. During physical handling, they kept an efficient posture, i.e., stretched arms and position of the lower extremities, and maintained an upright back position [47]. It is very important for nurses to have enough time for PH so that they can maximize the abilities of the patient and negate the limitations of the patient [2]. Based on our observations, we can assume that the majority of the observed nurses devoted enough time to patients because the PHI took place with a minimum of stress. As McMillan et al. [10] stated, to secure safe, high-quality physical handling both for the patient and nurses, it is necessary to use the right aids relative to the size and weight of the patient, to understand the type of PH needed, and to assess the patient´s level of independence as well as his/her general condition. The observed situations showed that out of 8 possible aids for PH listed on the observation sheet, nurses most often used a pad of bed linen (20 times) and set the bed to the correct height (26 times). The use of rollers (3 times) and slide sheets (5 times) was minimal. These two aids are very efficient since they decrease the load during handling and increase the patient´s comfort [12]. Situations that could not be performed by a single person were common. It is essential to call for assistance so as to complete the PH safely and carefully. Therefore, it is necessary to discuss the situation with colleagues and the patient and to determine how the handling will be done and who will do what. This is how most of the observed nurses routinely handled the problem. The observers assessed the PH as “suitable—cooperative, efficient; the nurse informs the assisting person and coordinates the planned steps; the nurse took preventive measures regarding possible harm to the patient and those assisting.” Nurses also showed that they understood the potential risks and they identified them in advance (e.g., adjustment of bed height, the function of the side rails and overhand pole, grip—how the patient uses the nurse for physical support, and level of patient cooperation). Most observers indicated the parameters. A few nurses expressed feelings of strain and/or pain either verbally i.e., “through an expression of effort, use of curse words, and loud interjections” or nonverbally by holding their back after the intervention, and by a movement suggesting an attempt to relax the overloaded back. Nonverbal expression was recorded more often.

## 4. Conclusions 

When comparing our results with research studies and other evidence, we can assume that the views of nurses and management regarding PH in nursing practice are comparable. We can say that there is not a big difference. However, the PH is not specified in detail in the documentation. The form and system of quality and safety concerning FH is not systematically organized. There is not much attention given to patient and nurse complaints. The perception and organization of physical handling by management is not clear when we consider unequal attitude towards PH. 

We found that the quality of care could be increased by utilization of a larger range of aids for PH, and more training focused specifically on PH. There was room for improvement relative to documentation, which should be specifically focused on covering the whole spectrum of possible handling issues, e.g., regarding body weight, the patient’s state of health and ability involved in the lack of homogeneity and consistency in the rules, methods, and documentation associated with PHI. Obtained results cannot be generalized, but they describe specific actual situations of observed samples in the Czech Republic. In conclusion, this issue needs more attention; it should be addressed by both those doing nursing research as well as those engaged in everyday nursing practice. Both will require the assistance of hospital staff and management. The top management of five hospitals in the Czech Republic’s South Bohemia region who are participants of the project are expecting the results to improve the quality of care and to prevent the risks related with PH.

### Limitations

We are aware of limitations. We take into account that the results could be influenced by the fact that the observations were made both by unit nurses and in some cases, by charge nurses and nurse managers who assisted with routine nursing care in their departments due to staffing shortages.

## Figures and Tables

**Table 1:** Category.

Category		Answer
1	Documents	“… *does not have anything like that: ... “We proceed according to the actual situation.“*
2	Form and system of quality and safety	“*It was not included.“; “… reporting and evaluation of adverse events (falls, pressure ulcers)”; “internal questionnaires investigating patient satisfaction…“*
3	Dealing with complaints and management	*“… reporting and evaluation of adverse events (falls, pressure ulcers)”; “internal questionnaires investigating patient satisfaction...“*
4	Perception and organization of physical handling by the management and education	*“… only employees who are interested in these lectures and want to listen to them apply and subsequently attend them (courses)...“*
5	Safety, risk and management.	*“… only employees who are interested in these lectures and want to listen to them apply and subsequently attend them... “*
